# Local Failure Rate in Oropharyngeal Carcinoma Patients Treated with Intensity-modulated Radiotherapy Without High-dose Clinical Target Volume

**DOI:** 10.7759/cureus.2958

**Published:** 2018-07-10

**Authors:** Hatim Almarzouki, Tamim Niazi, Michael Hier, Alex Mlynarek, Isabelle Lavoie, Khalil Sultanem

**Affiliations:** 1 Oncology, McGill University Health Center, Montreal, CAN; 2 Oncology, Jewish General Hospital, Montreal, CAN; 3 Otolaryngology, Jewish General Hospital, McGill University, Montreal, CAN; 4 Radiation Oncology, McGill University/Jewish General Hospital, Montreal, CAN

**Keywords:** local failure

## Abstract

Purpose: Our purpose was to evaluate whether omitting high-dose clinical target volume radiation (CTV-HD) around the gross tumor volume (GTV) in patients with oropharyngeal squamous cell carcinoma (OSCC) treated with intensity-modulated radiotherapy (IMRT) was associated with increased local failure.

Methods and materials: Patients diagnosed with stage I to stage IV OSCC between December 2004 and April 2017 were retrospectively reviewed. All patients were treated with radical radiotherapy using IMRT, with or without neoadjuvant or concurrent chemotherapy. In accordance with institution guidelines, CTV-HD was not used. Local failure was defined as disease persistence or reappearance at the primary tumor site. When primary failure was documented, the computed tomography/positron emission tomography (CT/PET) scan that showed primary failure was fused with the original treatment scan. Each recurrent tumor was contoured to evaluate the pattern of recurrence. Recurrences were categorized as in-field, marginal, or out-of-field if >95%, 20%-95%, or <20% of the recurrent tumor volume, respectively, was encompassed by the 95% high-dose prescription isodose line of the original treatment plan. We then determined whether omitting CTV-HD was associated with increased locoregional failure.

Results: A total of 272 patients with OSCC were assessed. The median follow-up from initial treatment was 43 months (range: 3-194 months). Seven patients were lost to follow-up. The overall five-year survival rate was 87%. The three- and five-year disease-free survival rates were 86% and 83%, respectively. Forty-one patients had 53 treatment failures (16 were local, eight were regional, and 29 were distant; some patients had treatment failures in multiple locations). Fourteen (87.5%) of the local recurrences were in-field, one (6.25%) was marginal, and one (6.25%) was out-of-field.

Conclusion: Our analysis of patients with oropharyngeal cancer suggests that local failure is mostly in-field and potentially due to radioresistance, rather than a marginal miss of the tumor. It suggests that omitting CTV-HD is feasible and safe.

## Introduction

Patients with advanced head and neck squamous cell carcinoma (HNSCC) are increasingly treated with radiotherapy, with or without chemotherapy, as the only curative nonsurgical treatment. This therapeutic strategy achieves high rates of local tumor control of over 80% for stages 1-2 and 60%–70% for stages 3-4 [1]. Recent advances in radiotherapy techniques, such as highly conformal intensity-modulated radiation treatment (IMRT) and image-guided radiotherapy (IGRT), have transformed the treatment of head and neck cancer by achieving both a reduction in long-term toxicity and better sparing of normal structures while effectively treating areas of tumor at risk [2].

The superiority of IMRT has been confirmed by randomized controlled trials, which have established IMRT as the current standard of care for the treatment of HNSCC [1,3]. However, with this technique, adequate delineation and coverage of target volumes become essential for local control [4]. In order to avoid a geometric miss of the tumor treatment area, it has become standard practice to add a high-dose clinical target volume (CTV-HD) around the gross tumor volume (GTV) [2,5-6]. This comes at the cost of increasing the volume of tissue receiving high-dose radiation and increases acute and late toxicities from treatment as well.

Local radiotherapy treatment failure can be related either to the radioresistance of the tumor or the geometric miss of the target tumor treatment volume [7]. The location of the recurrent tumor in relation to the original treated volume may indicate the cause of treatment failure. In the literature, the definition of a tumor recurrence location is done volumetrically, where the recurrence volume is compared with the 95% high-dose prescription isodose line of the original treatment plan and classified as (a) “in-field” if more than 95% of the recurrent tumor volume is within the 95% isodose line of the original treatment plan, (b) “out-of-field” if less than 20% of the recurrent volume is within this isodose line, or (c) “marginal” if between 20% and 95% of the recurrent tumor volume is within this isodose line [8-10]. In a majority of publications, in-field recurrence found inside the original high-dose target volume is assumed to indicate the radioresistance of the tumor, rather than a geometric miss of the tumor treatment volume [6,11-13]. In this retrospective study, we investigated whether or not omitting high-dose CTV was associated with a higher risk of geometric miss for local treatment failure.

## Materials and methods

Study design

A retrospective review was performed by examining patient charts, treatment plans, and diagnostic imaging studies of patients with oropharyngeal squamous cell carcinomas treated at our hospital. Patient consent was waived for reviewing patients' records and images by the institutional review board (IRB) of our hospital. Patients with histologically proven squamous cell carcinoma of the oropharynx, stage I to stage IV, using American Joint Committee on Cancer (AJCC) 7th edition, who had been treated with radical IMRT radiotherapy, with or without neoadjuvant or concurrent chemotherapy, were selected and their charts reviewed. Patients whose primary treatment modality was surgery were excluded. Patients who were treated with IMRT for recurrent disease or palliative radiotherapy, or who presented with distant metastases, were also excluded. When local treatment failure was documented, recurrence was assessed and delineated on post-recurrence computed tomography (CT) or CT/positron emission tomography (PET); then, the image was fused with the original treatment planning CT image, focusing on the area of recurrence. Failures were scored as in-field, marginal, or out-of-field if >95%, 20%-95% or <20% of the recurrent tumor volume, respectively, was encompassed by the 95% high-dose prescription isodose line of the original treatment plan. Imaging reports of recurrence and other available information, such as reports of physical examinations or fiberoptic laryngoscopy reports, were reviewed for recurrence delineation for patients with no available post-recurrence imaging.

## Results

Patient population

Between December 2004 and June 2017, 272 patients with histologically proven squamous cell carcinoma of the oropharynx, stage I to stage IV, were treated with radical radiotherapy, with or without concurrent chemotherapy, using an IMRT technique. We noted 139 patients with a tonsil primary tumor, 113 patients with a primary tumor at the base of the tongue, and 20 patients with a soft palate primary tumor. Clinical characteristics of our patient population are presented in Table 1.

**Table 1:** Patient characteristics M = male; F = female; HPV = human papillomavirus; T = tumor; N = node; RT = radiotherapy; CT = chemotherapy

Characteristics	Number	%
Age (year)		
<60	119	43.75%
>60	153	56.25%
Gender		
M	58	21.32%
F	214	78.68%
HPV		
Positive	168	61.76%
Negative	10	3.67%
Unknown	94	34.55%
T stage		
1	93	34.19%
2	84	30.88%
3	57	20.96%
4	37	13.60%
N stage		
1	47	17.28%
2a	37	13.60%
2b	135	49.63%
2c	40	14.71%
3	13	4.78%
Primary site		
Tonsil	139	41.46%
Base of tongue	113	56.09%
Soft palate	20	2.43%
Treatment		
RT alone	42	15.44%
RT + CT	230	84.55%

Treatment

All patients were immobilized with a thermoplastic head and neck mask and Accufix shoulder immobilization, and then they underwent computed tomography (CT) simulation scanning with 3-mm slices. Intravenous (IV) contrast was used in all patients. Diagnostic PET scan or magnetic resonance imaging (MRI) was fused with the planning CT scan from the original treatment. The attending physician defined the GTV, CTV, and organ at risk (OAR) volumes by contouring every CT slice. The GTV was defined to include the visible tumor and involved lymph nodes shown on CT, MRI, and/or PET imaging and thorough physical examination, including fiber-optic laryngoscopy. According to radiotherapy practice guidelines at our hospital, an accelerated hypofractionation schedule was used, with differential dose allocation to target volumes in a manner similar to the method described in Radiation Therapy Oncology Group (RTOG) protocols [4]. The GTV plus a 3-mm volume was labeled as the planning target volume (PTV), which was treated with a total dose of 67.5 Gy in 30 fractions of 2.25 Gy per fraction. The CTV was divided into two sub-volumes, CTV1 and CTV2. The GTV plus a margin of 0.5-1 cm and the involved lymph node region was designated CTV1. The remaining drainage lymph node regions were designated CTV2. A total of 60 Gy in 30 fractions was delivered to the periphery of the CTV1. The CTV2 was treated to a total of 54-56 Gy in 30 fractions. No high-dose CTV (CTV-HD = 67.5 Gy) was contoured, in accordance with institutional guidelines. Radiation dose planning was done initially using fixed beam IMRT, followed by a Volumetric Arc Modulated Therapy (Eclipse planning platform, Varian Medical Systems, Palo Alto, California, United States). Treatment was delivered using a Varian Linear Accelerator (Rapid Arc, Varian Medical Systems, Palo Alto, California, United States). The treatment was completed over six weeks using five daily fractions per week. Concomitant weekly chemotherapy with carboplatin (2-3 AUC) and Taxol (40 mg/m2) were used when needed [14-15].

Follow-up

The tumor response to treatment was assessed two to three months after completion. Evaluation included a clinical examination with fiberoptic laryngoscopy and CT imaging. The consideration of biopsy was based on clinical or radiological criteria. Patients were followed up with a physical examination every six to eight weeks during the first year after treatment, every three months for an additional two years, and then every six months until discharge at five years.

Local recurrence analysis

**Figure 1:**
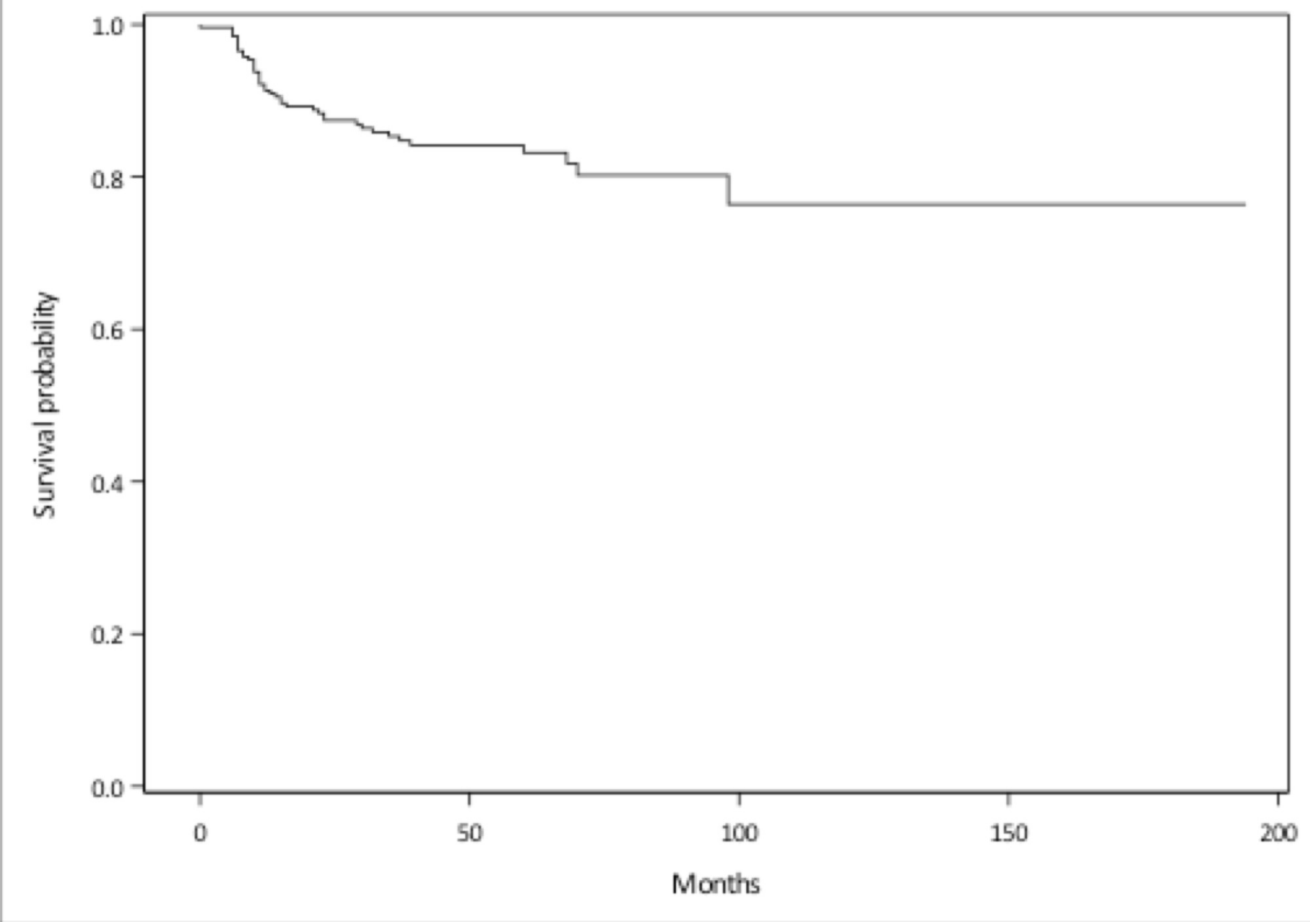
Disease-free survival

**Figure 2:**
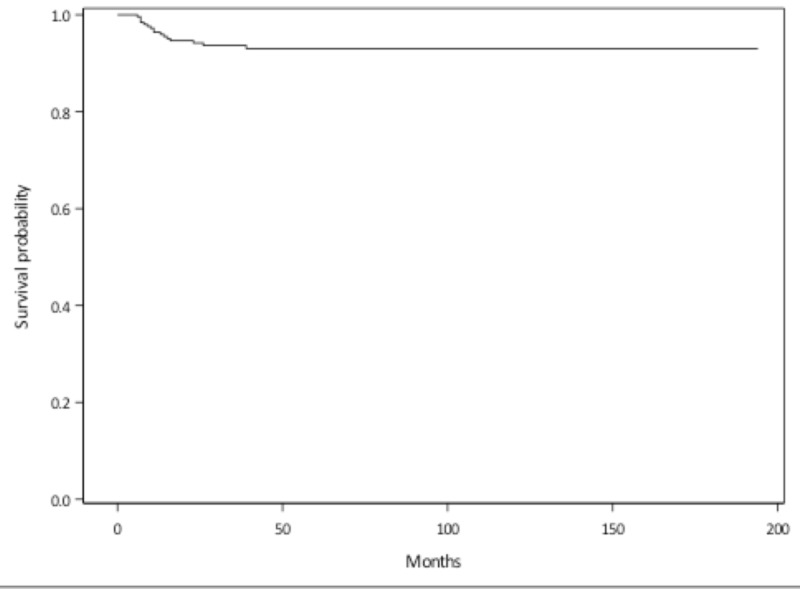
Local relapse-free survival

**Figure 3:**
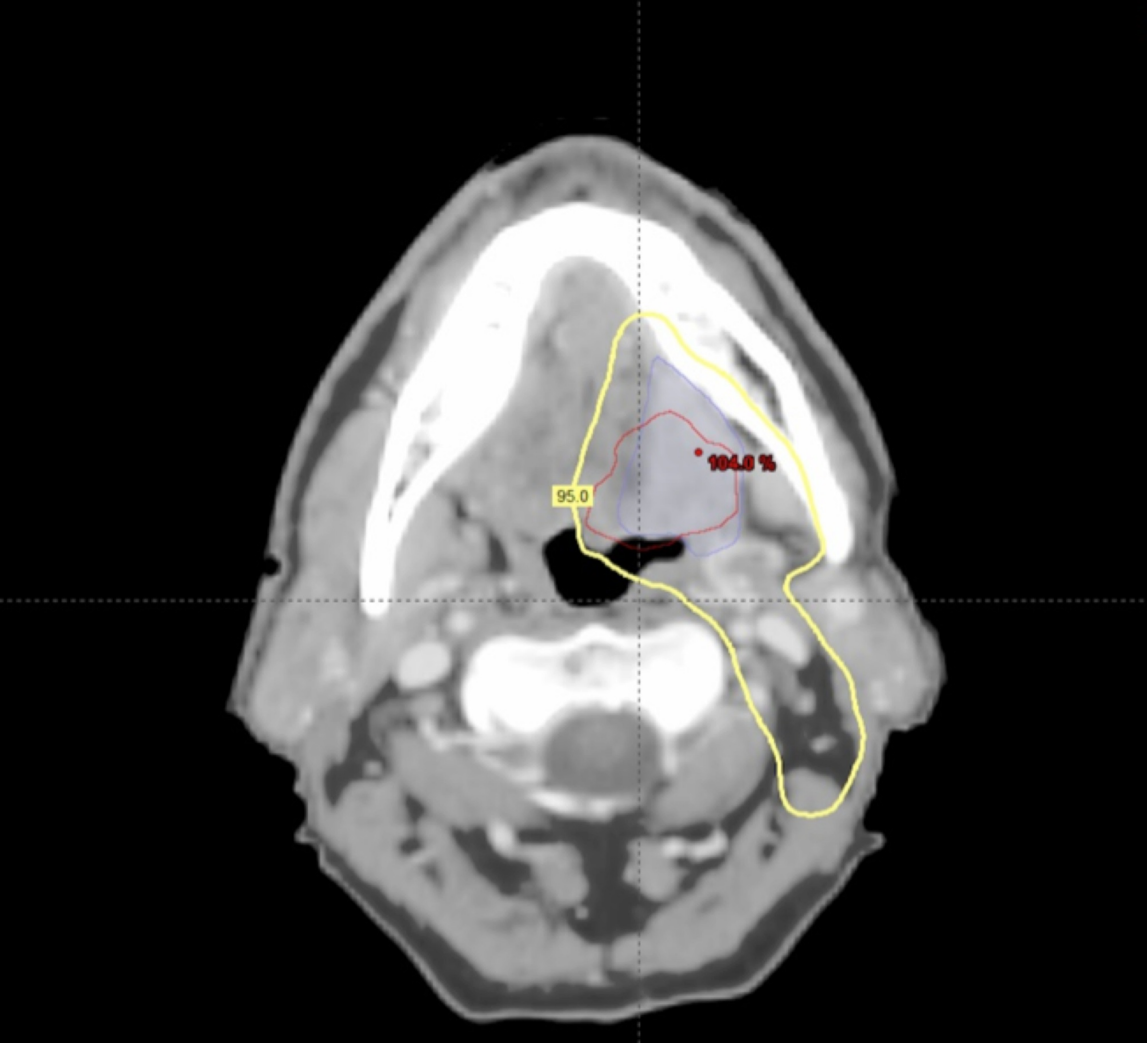
In-field recurrence T2 N1 carcinoma of the left tonsil treated with concurrent chemoradiotherapy; Original gross tumor volume (blue line); Delineation of recurrence (red line) after co-registration with original planning computed tomography; 95% isodose line (yellow).

**Figure 4:**
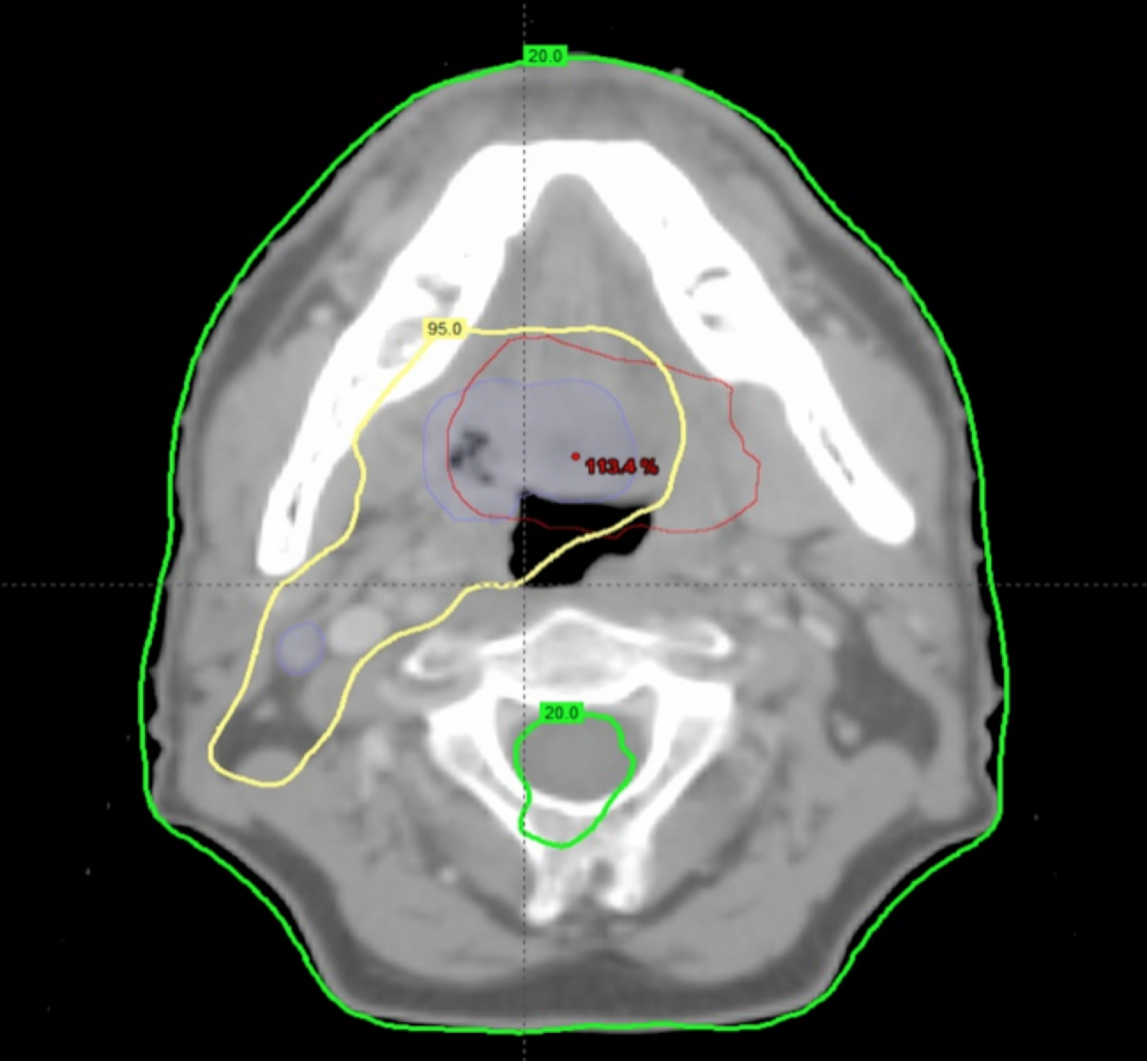
Marginal recurrence T2 N1 carcinoma of base of tongue treated with concurrent chemoradiotherapy; Original gross tumor volume (blue line); Delineation of recurrence (red line) after co-registration with original planning computed tomography; 95% isodose line (yellow); 20% isodose line (green).

A total of 272 patients with OSCC were assessed. Of this group, 139 patients had a tonsil primary tumor, 113 patients had a primary tumor at the base of the tongue, and 20 patients had a soft palate primary tumor. Median follow-up was 43 months (range 3-194 months). Seven patients were lost to follow-up. The five-year overall survival was 87%. The three- and five-year disease-free survival rates were 86% and 83%, respectively (Figure 1). In total, there were 41 patients with 53 initial treatment failures (some patients had treatment failures in multiple locations). Of all initial treatment failures, 16 were local, eight were regional, and 29 were distant (Figure 2). Of all local recurrences, 70% had imaging available for assessment. For the other 30% of the patients, clinical notes and radiology reports were used. We found that 14 (87%) of the local recurrences were in-field (Figure 3), one (6.5%) was marginal, and one (6.5%) was out-of-field. An example of in-field recurrence is shown in Figure 3 for T2 N1 carcinoma of the left tonsil treated with concurrent chemoradiotherapy. The marginal recurrence was in the base of the tongue, noted 16 months after primary treatment of a T2 N1 carcinoma treated with concurrent chemoradiotherapy (Figure 4).

## Discussion

Intensity-modulated radiotherapy has been adopted rapidly in the treatment of head and neck cancer, where accurate target volume definition is a key factor in the success of therapy, even though the steep penumbras of IMRT dose distributions, aimed at protecting radiosensitive normal tissue, initially raised some concerns about geometrical misses at tumor target borders [5,9]. However, in previous studies, the majority of local treatment failures originated in regions irradiated with high doses and seldom from the boundaries [7,10,16]. There is still no consensus on to how the high-dose CTV and PTV should be constructed. However, recommendations of the American Society of Therapeutic Radiology and Oncology (RTOG) have been published that emphasize anatomic expansions based on tumor site, size, and stage [17].

We are not aware of any systematic assessment that has been performed to determine the best (CTV-HD) margins, if any, to be added to the gross disease for IMRT treatments. Other authors have looked at the incidence and location of local failures as an indication of adequate target coverage. Eisbruch et al. found that in 133 patients, 17 of 21 recurrences were in-field (i.e., more than 95% of these volumes were within the 95% isodose line) [11]. Schoenfeld et al. found that 80% of the local recurrences were within PTV boundaries in 100 patients treated with IMRT [12]. Several other centers have reported failure patterns after IMRT [18-22]. Data from several large case series are summarized in Table 2, along with our experience, which showed comparable outcomes.

**Table 2:** Large series reporting patterns of failure using intensity-modulated radiotherapy for head and neck squamous cell carcinoma HNSCC = Head and neck squamous cell carcinoma

	Number of patients	Tumor site	Median follow-up (months)	In-field failures	Marginal failures	Out-of-field failures
Daly et al. [18]	107	Oropharynx	29	8	0	0
Garden et al. [19]	776	Oropharynx	54	77	7	5
Raktoe et al. [​​​​​7]	131	Oropharynx	40	35	0	4
Chao et al. [20]	126	HNSCC	26	17	3	0
Eisbruch et al. [11]	133	Non-nasopharyngeal	32	21	4	0
Yao et al. [21]	150	HNSCC	18	11	1	0
Studer et al. [22]	280	HNSCC	23	77	1	3
Schoenfeld et al. [12]	100	HNSCC	37	10	2	0
Jewish General Hospital, Montreal	272	HNSCC	43	14	1	1

Precise target volume delineation, both for improving treatment efficacy and for reducing radiation-induced toxicity, remains an area of active investigation. Identifying and integrating post-treatment imaging findings, such as site of failure, with the corresponding point in the original treatment plan, is essential to define high-risk areas and optimize guidelines for volume delineation. 

At our center, it is standard practice to omit CTV-HD radiation, in an attempt to minimize the high-dose treatment volume and the risk of acute and late toxicity-related complications. By performing a co-registration between the imaging of relapses and original treatment planning images in our study, we have shown that most treatment failures occurred predominantly in the GTV. In our study population, out of all local failures, only two patients were found to have a marginal or out-of-field failure, confirming that an appropriate target volume definition and adequate target dose coverage without the addition of a CTV-HD margin is safe and may lead to a possible reduction in acute and long-term radiation therapy-related toxicity. 

Limitations

One limitation of our study is its retrospective nature. In addition, at the time of recurrence, there may be significant changes in the external tumor contour of these patients due to factors, including patient weight loss and the changing shape of recurrent tumors. Also, in 30% of patients with local failure, the actual imaging was not available, and for these patients, we relied on clinical notes and radiology reports.

## Conclusions

Our analysis of patients with oropharyngeal cancer suggests that local failure is mostly in-field and potentially due to radioresistance, rather than a marginal miss of the tumor. It suggests that omitting CTV-HD is feasible and safe. Further validation of these findings is of interest in order to optimize volume definition guidelines in future head and neck cancer treatment protocols.
